# Understanding neuromuscular disorders in chronic fatigue syndrome

**DOI:** 10.12688/f1000research.18660.1

**Published:** 2019-11-28

**Authors:** Yves Jammes, Frédérique Retornaz

**Affiliations:** 1 C2VN Inserm Inra, Faculty of Medicine, Aix Marseille University, Marseille, France, France; 2 Department of Internal Medicine, European Hospital, Marseille, France, France

**Keywords:** myalgic encephalomyelitis, chronic fatigue syndrome, central fatigue, peripheral fatigue, oxidative stress, heat shock proteins, neurophysiology, physiology, neuromuscular disorders

## Abstract

Muscle failure has been demonstrated in patients with myalgic encephalomyelitis/chronic fatigue syndrome (ME/CFS). Neurophysiological tools demonstrate the existence of both central and peripheral fatigue in these patients. Central fatigue is deduced from the reduced amplitude of myopotentials evoked by transcranial magnetic stimulation of the motor cortex as well as by the muscle response to interpolated twitches during sustained fatiguing efforts. An impaired muscle membrane conduction velocity assessed by the reduced amplitude and lengthened duration of myopotentials evoked by direct muscle stimulation is the defining feature of peripheral fatigue. Some patients with ME/CFS show an increased oxidative stress response to exercise. The formation of lipid hydroperoxides in the sarcolemma, which alters ionic fluxes, could explain the reduction of muscle membrane excitability and potassium outflow often measured in these patients. In patients with ME/CFS, the formation of heat shock proteins (HSPs) is also reduced. Because HSPs protect muscle cells against the deleterious effects of reactive oxygen species, the lack of their production could explain the augmented oxidative stress and the consecutive alterations of myopotentials which could open a way for future treatment of ME/CFS.

## Introduction

Chronic fatigue syndrome (CFS), also called myalgic encephalomyelitis/CFS (ME/CFS), is a multisystem disease with immune dysfunction and autonomic abnormalities characterized by an intense fatigue worsened by physical/mental activity ^1, 2^. It is often associated with post-exertional malaise (PEM) ^2, 3^. Its pathogenesis appears to have a number of factors; different stressors (such as physical exertion, severe infections, or emotional stress or a combination of these) are continually reported in the medical history of patients with ME/CFS ^4^. An altered skeletal muscle function has been observed in ME/CFS pathogenesis ^5– 9^. In our studies ^5– 8^, alterations of the muscle membrane excitability in response to exercise was found in 86 out of 133 patients with ME/CFS (that is, 65%). Several ME/CFS studies have also reported an enhanced oxidative stress in response to exercise ^4– 7^. This mini-review focuses on the neurophysiological disorders found in patients with ME/CFS and changes in biochemical markers of exercise, such as the potassium outflow, oxidative stress, and heat shock protein (HSP) response.

## The general mechanisms of muscle fatigue

Muscle fatigue results primarily from the incapacity of the muscle fibers to contract. Muscle failure called “peripheral fatigue” may result from a failure of different metabolic processes such as the imbalance between oxygen demand and supply, the reduced excitation–contraction coupling involving altered intracellular calcium release and mobilization, and the impaired muscle membrane excitability due to the altered flux of potassium through the sarcolemma ^10^. “Peripheral fatigue” is generally preceded by the reduced recruitment of motoneurons which drive the highly fatiguing motor units. This phenomenon, called “central fatigue”, tends to delay the occurrence of “peripheral fatigue” (the “muscle wisdom” phenomenon). In humans, non-invasive tools are used to explore “peripheral” and “central” fatigue. Peripheral fatigue is assessed by the reduction of the contractile response (twitch) to direct electrical muscle stimulation. On the other hand, central fatigue is present when the interpolation of twitches elicited by repetitive electrical muscle stimulation or transcranial magnetic stimulation (TMS) of cortical motor areas restores a contractile response during fatiguing efforts. Muscle fatigue is closely linked to an excessive production of reactive oxygen species (ROS) ^11^. The sensory pathways carried by the group III and IV muscle afferents play key reflex roles in triggering the muscle wisdom phenomenon. The motor drive of both working and resting muscles is modulated by these muscle afferents through their spinal and supraspinal projections and their afferent pathways, supporting the sensation of muscle fatigue and pain ^12, 13^. Multiple stressors, such as fatiguing muscle contraction, muscle acidosis, hypoxia, ischemia, and ROS, stimulate these muscle afferents ^14– 16^. Their activation by muscle fatigue triggers the widespread production of HSPs ^17^.

## In patients with myalgic encephalomyelitis/chronic fatigue syndrome, central and peripheral fatigue coexist

### Central fatigue

Some physiological studies using the twitch interpolation technique and analyzing the maximal voluntary contraction cannot support the hypothesis of central fatigue in patients with ME/CFS ^18, 19^. By contrast, numerous studies support the existence of central fatigue in these patients. Kent-Braun *et al*. ^20^ showed that the voluntary contraction of the tibialis muscle during maximal isometric exercise was lowered. In patients with post-infectious CFS, Sacco *et al*. ^21^ reported a reduced amplitude of motor potentials evoked by TMS of the motor cortex in the biceps brachii muscle. The authors also reported an increased interpolated twitch amplitude during sustained fatiguing efforts in patients with ME/CFS. The same observations were made by Schillings *et al*. ^22^. Davey *et al*. ^23^ correlated day-to-day changes in ME/CFS symptomatology with the changes in simple reaction times (SRTs) and movement times of myopotentials evoked in muscles by TMS of the motor cortex, and corticospinal excitability was assessed by measuring the threshold TMS intensity. The authors reported slowed SRTs and increased threshold intensity, supporting the existence of a deficit in motor preparatory cortical areas. Siemionow *et al*. ^24^ reported a modification of the central motor command to muscles during isometric handgrip and measured an increased relative power of electroencephalography theta frequency band in patients with ME/CFS compared with healthy volunteers. These observations suggest that ME/CFS pathology may be associated with an altered central nervous system command to muscles.

Perception of effort and pain seems to be accentuated in patients with ME/CFS. This was previously reported by Sacco *et al*. ^21^ and more recently confirmed ^25^. The group III or IV metabosensitive muscle afferents present in all skeletal muscles are strongly activated by the oxygen free radicals ^16^, a situation amplified in patients with ME/CFS ^5, 6^. It is tempting to speculate that increased activation of muscle afferents in patients with ME/CFS could result in an accentuated perception of effort and pain (myalgia). The key role played by these muscle afferents in central fatigue (muscle wisdom phenomenon) could also explain the numerous observations of a diminished central activation, documented in ME/CFS ^20– 24^.

### Peripheral fatigue

Delayed recovery from fatiguing exercise in patients with ME/CFS may be due to peripheral muscle fatigue ^9, 26^. During incremental cycling leg exercise approaching the maximal oxygen uptake (VO _2_), marked alterations of myopotentials in response to direct muscle stimulation (M-wave) have been observed in a number of patients with ME/CFS ^5– 8^. These M-wave changes began early in exercise and culminated at the end of a 30-min recovery. This suggests the existence of peripheral fatigue due to impaired muscle membrane excitability. Similar M-wave alterations are absent in healthy subjects, for whom the amplitude of myopotentials either does not vary or even increases with the incremental pedaling force ^5^.

## Biological events accompanying the electrophysiological disorders

### Reduced ionic fluxes through the muscle membrane

Alteration of ionic fluxes through the sarcolemma could explain the altered muscle membrane excitability reported in patients with ME/CFS. In healthy subjects, muscle biopsies demonstrated a physiological contraction-induced loss in myoplasmic potassium (K ^+^) concentration ^27^. This potassium outflow is detectable in plasma, and the kinetics of plasma K ^+^ increase during and after an incremental exercise is well known ^28^. A study by Fulle *et al*. ^29^ confirmed the presence of alterations in ryanodine channels and a deregulation of Na ^+^/K ^+^ and Ca ^2+^-ATPase pumps in the membranes of sarcoplasmic reticulum in patients with ME/CFS. To explain their data, Fulle *et al*. ^30^ suggested that the deregulated pump activities could result from an increased fluidity of the sarcoplasmic reticulum membrane in these patients.

### Increased production of reactive oxygen species

Several studies in patients with ME/CFS have examined changes in resting blood oxidant–anti-oxidant status and reported lower vitamin E concentration and higher levels of oxidized LDL, thiobarbituric acid reactive substances (TBARS), and malondialdehyde (MAL) ^31– 33^. In biopsies of vastus lateralis muscle of patients with ME/CFS, Fulle *et al*. ^30^ detected oxidative damage to DNA and lipids and increased activity of intracellular anti-oxidants (catalase, glutathione peroxidase, and transferase). Other authors also found a correlation between musculoskeletal symptoms and an accentuated lipid peroxidation at rest in patients with ME/CFS ^33, 34^. Plasma markers of oxidative stress are the TBARS, a marker of lipid peroxidation, and reduced ascorbic acid, an endogenous anti-oxidant ^5– 7, 31– 34^.

In healthy subjects, exercise induces modest oxidative stress ^5, 35, 36^, whereas marked exercise-induced production of ROS has been found in patients with ME/CFS ^5– 7^. The muscle production of oxygen free radicals is proportional to that of VO _2_^35, 36^. From several reports ^5, 37, 38^, VO _2_ measurement in exercising patients with ME/CFS indicated a normal aerobic function; indeed, their maximal VO _2_ was in the normal range. However, a recent study ^39^ showed that, perhaps because of PEM, patients with ME/CFS were unable to reproduce cardiopulmonary exercise testing during a second test. An *in vitro* study in skeletal muscle cell culture ^40^ showed that, after electrical pulse stimulation mimicking PEM, patients with ME/CFS, compared with normal subjects, had no increase in AMPK phosphorylation, a defect of glucose uptake, and a reduction of interleukin-6 (IL-6) secretion, highlighting the reality of lowered metabolic performance of muscle cells during PEM. A recent study by Richardson *et al*. ^41^ proposed using the weighted standing time as a proxy for PEM severity in patients with ME/CFS.

An inhibitory action on Na ^+^-K ^+^ pump activity is exerted by increased production of ROS during exercise ^11^ and this reduces muscle membrane excitability and potassium outflow. Published ^5– 8^ and unpublished observations have noted that the magnitude of altered muscle membrane excitability (reduced M-wave amplitude) is proportional to the reduction of exercise-induced potassium outflow and to the magnitude of oxidative stress in patients with ME/CFS. Moreover, in 42% of the 69 patients with ME/CFS, PEM was associated with post-exercise alterations of muscle membrane excitability.

### Reduced heat shock protein production/expression

The HSPs protect cells against the deleterious effects of ROS produced during exercise ^42, 43^, reducing the generation of ROS through the activation of anti-oxidants. The oxidant levels, in turn, increase the level of plasma HSP ^43^. In patients with ME/CFS, the responses of plasma HSP27 and HSP70 to exercise can be delayed and often reduced, and resting levels of plasma HSP70 are lower in these patients than in healthy volunteers ^6^. The lack of HSP response to exercise might explain the augmented oxidative stress measured in these patients. As already suggested ^7^, a downregulation of HSP production in some individuals could be caused by the repetition of exercise bouts at high energetic levels. As cited above, the activation of the group III or IV muscle afferents triggers the HSP production in working and resting muscles as well as in the brain and different organs ^17^. It may be hypothesized that the prolonged activation of these muscle afferents by the oxidative stress could induce a reduction of HSP production in patients with ME/CFS. Further studies, including in high-intensity sport programs and military training, are needed to show that the repetition of exercise bouts at high levels might depress the expression of the inducible factors of HSP However, HSP malfunction was also reported in different pathologies and may have origins other than the repetition of stressors. Thus, in patients with multiple sclerosis and systemic lupus erythematosus, Elfaitouri *et al*. ^44^ measured an IgM to specific cross-reactive epitopes of human HSP60 compatible with infection-induced autoimmunity. HSP dysfunction was also reported in patients with chronic fatigue in primary Sjögren’s syndrome ^45^. Because antibodies to a microbial HSP60 may cross-react with human HSP60 ^46^, it may be that infectious diseases often reported in patients with ME/CFS alter their HSP function.

## The role played by history of severe infections in the neuromuscular disorders of patients with myalgic encephalomyelitis/chronic fatigue syndrome

In a previous study ^6^, it was reported that the history of infection in patients with ME/CFS was associated with a marked significant increase in M-wave alterations and a reduced exercise-induced potassium efflux. The post-exercise changes in M-wave amplitude were correlated to a significant reduction of the maximal potassium outflow measured at the end of the exercise and to the baseline TBARS level. A further study highlights the importance of infectious stressors in ME/CFS pathogenesis and biological expression. A significant reduction of muscle excitability during work and increased blood oxidant status disorders at rest were measured in ME/CFS patients who reported a recent severe infection due to H1N1 influenza, pneumonia, encephalomyelitis, or sepsis ^7^. It is well documented that acute infection constitutes a trigger for an oxidative stress ^46– 48^. A review by Rasa *et al*. ^49^ compiles all of the studies carried out so far to investigate various viral agents that could be associated with ME/CFS. However, the role played by viral infection in ME/CFS pathogenesis is not clear. Recent observations by Bouquet *et al*. ^50^ do not support immune cell dysregulation or viral reactivation in ME/CFS patients after exercise bouts inducing PEM.

## Conclusions

This review focuses on the neurophysiological modifications that associate central and peripheral fatigue, reduced potassium outflow from exercising muscles, altered equilibrium between pro- and anti-oxidants, and a reduced expression/production of HSPs in patients with ME/CFS. A mechanistic approach to the causes of neurobiological disorders in the ME/CFS pathology is proposed on the basis of a reduction in the protective role of HSP. Repeated and combined stressors (high exercise level, infections and perhaps also psychological stress) in the history of these patients might contribute to a depletion of HSP production or its expression or both. The consequences of a dysregulation of the oxidant/anti-oxidant status might result both in an altered muscle membrane excitability (peripheral fatigue) and in an augmented activation of the group III or IV muscle afferents which play a key role in the mechanism of central fatigue. Correcting any deficiency in HSP production could open a future way for the treatment of ME/CFS.

## Abbreviations

HSP, heat shock protein; ME/CFS, myalgic encephalomyelitis/chronic fatigue syndrome; M-wave, muscle action potential; PEM, post-exertional malaise; ROS, reactive oxygen species; SRT, simple reaction time; TBARS, thiobarbituric acid reactive substances; TMS, transcranial magnetic stimulation; VO _2_, oxygen uptake
